# Towards a Solution to the Goose-Agriculture Conflict in North Norway, 1988–2012: The Interplay between Policy, Stakeholder Influence and Goose Population Dynamics

**DOI:** 10.1371/journal.pone.0071912

**Published:** 2013-08-20

**Authors:** Ingunn M. Tombre, Einar Eythórsson, Jesper Madsen

**Affiliations:** 1 Norwegian Institute for Nature Research, Arctic Ecology Department, The Fram Centre, Tromsø, Norway; 2 Norwegian Institute for Cultural Heritage Research, The Fram Centre, Tromsø, Norway; 3 Aarhus University, Department of Bioscience, Arctic Research Centre, Aarhus, Denmark; Cairo University, Egypt

## Abstract

This paper presents results from a multidisciplinary study of a negotiation process between farmers and wildlife authorities which led to an agricultural subsidy scheme to alleviate conflicts between agriculture and geese in Norway. The Svalbard-breeding population of pink-footed geese *Anser brachyrhynchus* has increased considerably over the last decades and conflicts with farmers have escalated, especially at stopover sites in spring when geese feed on newly sprouted pasture grass. In Vesterålen, an important stopover site for geese in North Norway, farmers deployed scaring of geese at varying intensity dependent on the level of conflict during 1988–2012. We assessed the efficiency of a subsidy scheme established in 2006, in terms of its conflict mitigation, reflected in a near discontinuation of scaring activities. The presence of pink-footed geese was analysed in relation to scaring intensity, the total goose population size and the increasing occurrence of another goose species, the barnacle goose *Branta leucopsis*. Scaring significantly affected the number of geese staging in Vesterålen, both in absolute and relative terms (controlling for total population size). The geese responded immediately to an increased, and reduced, level of scaring. Despite the establishment of the subsidy scheme, the number of pink-footed geese has recently declined which is probably caused by the increasing number of barnacle geese. For the farmers, the subsidy scheme provides funding that reduces the economic costs caused by the geese. Sustaining a low level of conflict will require close monitoring, dialogue and adaptation of the subsidy scheme to cater for changes in goose population dynamics.

## Introduction

Most of the temperate-wintering goose populations in Europe have increased in numbers due to a combination of protection, establishment of nature reserves at the breeding grounds and improved feeding opportunities in the wintering areas where the geese have expanded their use of farmland areas [1], [2], [3]. Advances in farming technology, increased use of fertilizers and changes in crop types provide geese with larger fields of higher yielding food [4], [1], [5], [6]. The increasing goose populations cause serious conflicts with agriculture by reducing the harvest or direct competition with livestock [1], [7], [8], [9], [10], [11]. For northwest Europe, an intensification of the conflict is expected for several future land-use scenarios [12], especially since several goose populations continue to grow [2].

For wildlife management, these conflicts are challenging. Nyhus and co-workers [13] noted that “the issues surrounding such conflicts are typically a complex mix of behaviour (human and wildlife), ecology, socio-economics, politics and geography, making the resolutions of these conflicts extremely difficult”. In the case of damage to agricultural crops caused by wild geese, the impact is often geographically concentrated and represents an uneven burden on farmers in the most affected areas.

In Scotland, local compensation schemes, funded by the Scottish Natural Heritage, have evolved since the 1990’s [14], [15], [16], [17]. Within an overall national policy framework, schemes tailored to local conditions and stakeholder input at all levels have been crucial for the success [15]. A combination of grassland managed and allocated for geese, areas with scaring and shooting and subsidized areas with payments to farmers have been employed. For instance, the population of Svalbard-breeding barnacle geese *Branta leucopsis* winters in a rather restricted area in Solway Firth, UK. The establishment of a payment scheme to farmers with subsidized areas in order to reduce the level of goose scaring has lead to acceptable conditions both for the agricultural practice in the region and the wintering goose population [14]. In the Netherlands, geese responded to an implementation of a large-scale non-disturbance policy, where years without scaring involved an increase in the carrying capacity of natural, not cultivated, habitats; more geese used the sites for a longer period [18]. In general, however, management of the goose-agriculture conflict has been dealt with at the local level on an *ad hoc* basis [9]. In areas where no management or schemes are established, scaring is in most cases the only means available to farmers to defend their crops. However, even if the species in question are not listed as endangered, scaring may be questioned on ethical grounds. Moreover, organised scaring is time-consuming [10], and often requires co-ordination among neighbours [19]. Furthermore, applying scaring as the only means is only likely to ease goose problems in one area at the expense of another, as the geese move on to alternative feeding grounds [20].

From a social fairness perspective, it is unreasonable that individual farmers have to pay the cost of feeding growing populations of geese. This does not necessarily mean that all goose grazing on agricultural land should be compensated, but the use of compensation to alleviate the conflict has become an urgent issue in areas where large concentrations of grazing geese have become a serious problem for agriculture as a result of the expanding goose populations. Agri-environment schemes, where farmers are financially compensated in order to modify their farming practices in an advantageous environmental direction (e.g. accept spring staging geese on their properties), have been introduced in several countries in Europe [14], [21], [22]. The positive effects of many of these actions have, however, been difficult to demonstrate since the design of these initiatives were inadequate to reliably assess their effectiveness [22]. Moreover, where the effects of the schemes were possible to measure only half of them had positive consequences for the environment in terms of, for instance, increased biodiversity [22], [23]. Whether there were any positive effects in terms of conflict alleviation is unknown.

There are a number of contested issues regarding the form and amount of compensation; which government budget should come from, who should be entitled to it and on what grounds? In principle, a fair compensation should reflect the economic loss suffered by each individual farmer, but in practice, annual verification and evaluation of damage on each affected farm is complicated and expensive.

In this paper, we present results from the process which led to the subsidy scheme for spring-migrating arctic geese in Norway. We assess the consequences for geese and farmers after it was implemented and define success, in terms of goose management, as an outcome which a) secures acceptable conditions for the goose populations, b) provides an acceptable compensation for damage on agricultural crops and c) alleviates the conflict between wildlife and agriculture. In the Norwegian case, the process of designing and implementing a subsidy-scheme for goose has shown that conflict resolution requires an efficient collaboration between different branches of government (environment and agriculture), as well as real and clearly defined stakeholder involvement in the process of planning and decision-making [19]. Other studies on wildlife-agriculture conflicts point in the same direction. Hence, an integrated approach, with clearly defined objectives amongst different stakeholder groups, maintaining the communication among the different parties of the conflict, as well as a realisation of the fact that local people often have a reciprocal interest in the resources in question, has been shown to be vital in order to successfully achieve management goals [14], [24], [25], [26], [16], [17].

The Svalbard-breeding population of pink-footed geese *Anser brachyrhynchus* migrates via Norway in spring, and Vesterålen North Norway is an important stopover site [3], [27]. After departing Vesterålen, they migrate more than 900 km further north to the high-arctic Svalbard breeding grounds. In recent years, also barnacle geese from the Svalbard-breeding population have made a stopover in the area [28], [20], [unpublished data]. Both populations have increased substantially in recent decades, especially the population of pink-footed geese [29]. The grazing area in Vesterålen is limited, and most of the available habitats are managed grassland [30], [7]. From the early 1990s to 2004, there was an intensive conflict between agricultural interests and geese in this region [19], [31], [20]. Since then, a subsidy scheme has been introduced. In the present study, we describe this conflict and the process towards a management scheme to mitigate the conflict. We demonstrate how this process, resulting in varying intensities of scaring over the years, influenced the number of staging geese in the region. By following different *phases* of conflict and negotiation in the Vesterålen case, we substantiate that *scaring intensity* can be used as an indicator of *conflict level* during each phase. The objective of the analysis is to evaluate the effects of scaring intensity on the amount of geese in the area, represented by *goosedays*, as registered by biological research through different phases of conflict and negotiations over the last decades.

## Materials and Methods

### Study Area and Study Populations

The agriculture in Vesterålen (68°38′N, 14°20′E), North Norway, is dominated by grasslands used for cattle and sheep grazing and feeding. Most of the farmers in the study area have other paid employment in addition to being a farmer (Statistics Norway, http://www.ssb.no/kommuner/jordbruk/). The geese feed on farmland in close vicinity to the coast (<1 km), and experience 24 hours of daylight during most of their staging period in May. Hence, they can potentially feed day and night. During daytime, geese mostly forage in outer fields; whereas, at night when there is less human disturbance, the geese move close to roads and buildings [30]. The population size of pink-footed geese has increased from c. 20,000 in the 1970s to a hitherto unprecedented peak of around 80,000 in 2012 [29]. The population spends the winter and early spring in Belgium, The Netherlands and Denmark [27], and main spring staging areas in Norway are at two specific sites; Nord-Trøndelag in central Norway and Vesterålen in North Norway. The barnacle goose population has grown from around 10 000 in the 1970s to approximately 33 000 individuals at present [32], [33]. The population spends the winter in the UK and migrates in spring to Svalbard via Helgeland in Mid-Norway. Spring range has expanded northwards in recent decades [34], [28], and the proportion of the population staging in Vesterålen has increased significantly over the last fifteen years [35]. At present, 27% of the population use Vesterålen in spring [28]. The importance of Vesterålen for spring-staging geese as a critical site building up energy reserves before breeding where individuals with extended use of Vesterålen, as compared to those staging at the site further south, were most successful in terms of reproductive output [36]. The increase of both goose species in the region has escalated the conflicts with agriculture over the years [19], [31], [37], [20].

### Methods

A combination of biological and social science methods were applied for the purpose of an analysis of the relationships between changes in management policy, variation in the intensity of scaring activity by farmers and the number of staging geese during 1988–2012. The intensity of scaring activities has varied at different stages of the process, and can be seen as a reflection of conflict levels during different phases of a process of negotiations for a management policy.

### Assessment of Conflict Level at Different Stages

Data on the process of conflict, planning, negotiation and policy-making for goose management were collected through interviews and document studies. A series of interviews with participants/stakeholders was carried out in 2001 and 2002. This series included a small number of local farmers (seven), an agricultural official from the municipality with the most geese by that time (Sortland), one regional representative each from the Norwegian Farmers Association and the Norwegian Association of Farmers and Smallholders, a local branch of the Norwegian Ornithological Society, the Norwegian Institute for Nature Research (one researcher), the Department for Environmental Protection and the Agricultural Department at the County Governoŕs Office in Nordland (a wildlife manager and an agronomist at the regional level) and the Directorate for Nature Management (a manager at the national level). A second series of interviews involving 10 farmers and 2 municipal officers was conducted in 2012. For these kind of interviews, ethics approval was not necessary based on the Norwegian Law 2000-04-14 no. 13 (“*Law on personal information § 1–52”*). According to this law (§ 31) a notification to *The Norwegian Data Protection Authority* is required a) in cases where personal data are processed electronically, or b) if a manual register of persons containing sensitive personal data is established. The laws § 2, section 1 defines *personal data* as information and assessments that can be traced to an individual. The same paragraph, section 8 defines *sensitive personal data* as information about race or ethnicity, political, philosophical or religious conviction, criminal record, health, sexual issues or union membership. In the research project on which this paper is based, no sensitive personal data are collected. The data collected through interviews have not been processed electronically. The interview data are treated anonymously and cannot be traced back to individuals, and are thus not personal data according to the above quoted legal definition. All the persons were verbally agreed to participate in the study and were more than willing to share their experience and opinions with the researchers. This process was documented via notebooks.

Written sources, in the form of government reports, research reports, plan documents, letters, resolutions and minutes from meetings were also important data sources. The conflict level varied at different stages in the process, depending on perceived progress or setbacks in the dialogue between the involved actors. Hence, to a certain degree, intensity of scaring activities can be interpreted as a reflection of conflict level at different point of time during the process. Based on these data, we have identified 6 phases which represent different intensities of scaring (0, 1, 2, see definitions below), linked to different levels of conflict throughout the period of study; Phase 1 with scaring intensity 0 (1988–1992), Phase 2 with scaring intensity 1 (1993–1995), Phase 3 with scaring intensity 0 (1996–1998), Phase 4 with scaring intensity 2 (1999–2003), Phase 5 with scaring intensity 1 (2004–2006) and Phase 6 with scaring intensity 0 (2007–2012).

### Registration of Scaring Activity

During daily goose monitoring carried out in Vesterålen since 1988 (see below), scaring activities by farmers were recorded whenever observed. All observations were conducted from official roads or sometimes at the edge of the different fields. Whenever using private paths or tracks, the landowner was asked in advance and normally a general permission (“as long as you want”) was given. Many farmers announced in advance of each season that they were planning to chase the geese off their properties. Hence, along with information from local agricultural officers, the pattern of scaring was well known for each season. Moreover, after the subsidy scheme was established, there were no scaring activities on the fields participating in the scheme (one of the premises for attending). The scaring activity each year was classified in three levels (equivalent to classification used in 31); 0 = no scaring, 1 = moderate scaring practiced at some locations in the region, and 2 = intensive and systematic scaring organised at most of the goose sites.

### Goose Registrations

Systematic goose registrations have been carried out annually in the Vesterålen region since 1988. Professionals and trained amateur observers counted geese from cars and vantage points in the terrain using telescopes and binocular. The researchers never interacted with the geese in any way, but were observed from a distance. One of the species, the barnacle goose, is a protected species. The goose registrations were carried out in the period from late April/early May to late May (around the 22^nd^). Daily goose counts are summarised from a 14 day core-period for the whole region, from the 7^th^ to the 20^th^ of May, covering the main spring staging period. The number of goose days per year, over a period of 14 days, was calculated following the equation.
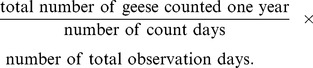



This gives comparable figures between years accounting for varying numbers of count days.

The estimation of total population size of pink-footed geese is based on co-ordinated wintering surveys in Denmark, the Netherlands and Belgium [38], [39] supported by co-ordinated spring counts along the flyway. The number of pink-footed geese using Vesterålen in spring may be a reflection of the total population size, that is, more pink-footed geese in the area may simply be a consequence of a larger population size. Hence, in order to control for this, a relative measure (for the observation days 7–20 May) was used as a yearly average following the equation.
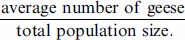



### Statistical Data Analyses

In analyses evaluating how the relative goose number changed over years, a linear regression was conducted for the whole study period and for the different phases with different scaring regimes with separate ANOVAs. We analysed the variation in relative numbers of pink-footed geese using mixed models (PROC MIXED) with normal error and identity link functions. We considered phase (1–6), scaring activity by farmers (0, 1, 2) and the occurrence of barnacle geese in relation to pink-footed geese (averages of daily counts per year of barnacle geese divided by the corresponding number of pink-footed geese) as fixed effects. Statistical tests were carried out using the statistical package SAS [37]. P-values less than 0.05 were considered significant.

## Results

### The Process towards a Subsidy Scheme

**Figure 1:**
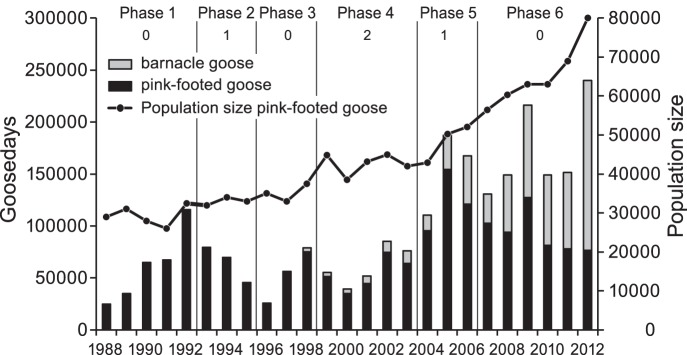
The population size of pink-footed goose, and the development of goosedays in relation to different phases of management and scaring. The number of goosedays per year in Vesterålen, North Norway, where black bars represent pink-footed geese *Anser brachyrhynchus* and grey bars represent barnacle geese *Branta leucopsis*. The curve is the total population size of pink-footed geese (right axis). Six different phases, classification described in methods, are indicated. 0, 1 and 2 represent intensities of scaring, intermediate scaring and intensive scaring, respectively. The goosedays calculations are described in methods. We performed separate ANOVAs for each phase and scaring regime to measure changes in goosedays (see results for values).

The goose scaring started in Vesterålen in 1993 (Phase 2 in Fig. 1) when farmers from the most affected areas decided to co-ordinate their scaring efforts on a neighbourhood basis. At the same time, local branches of the Norwegian Farmers’ Union and the Norwegian Farmers and Smallholders Union took initiatives to bring the conflict on the wildlife management agenda. According to one of the most active farmers in this process [19], the organised scaring regime served a double purpose; to prevent damage on farmland and to put pressure on the Directorate for Nature Management (DN). In 1994, DN responded by inviting The Ministry of Agriculture, The Norwegian Farmers’ Union, The Norwegian Farmers and Smallholders Union, The Norwegian Association of Hunters and Anglers, The Norwegian Ornithological Society, The Governor of Svalbard and The Norwegian Institute for Nature Research to participate in the preparation of an “Action Plan for Goose Management”. Despite some internal differences, the plan group produced a comprehensive plan document which was launched in 1996 [41]. The plan included proposals to solve the goose-agriculture conflict by administrative and economic means, within a framework of local management plans, worked out in collaboration between wildlife- and agriculture authorities and stakeholders. With the prospects of imminent solution, the Sortland farmers agreed to stop the scaring in 1996 (Phase 3 in Fig. 1). In Sortland, a local management plan was developed, with participation from farmers and the ornithological society. The Sortland management plan, which was completed in 1997 [42], concluded that in addition to other measures, annual compensation to affected farmers was a necessary part of a solution to the goose-agriculture conflict. The National Action Plan was vague in terms of what kind of compensation should be applied, and the Sortland plan was not approved since the DN was not willing to fund monetary compensation for goose grazing. In 1999, the farmers had lost their patience with DN and initiated a systematic and organised scaring campaign, involving more farmers in a 24 hours scaring regime. The political protest was a strong motivation for participation in the scaring regime; the disappointment and loss of confidence in the management institutions reinforced farmerś solidarity and motivations. Intensive goose scaring was maintained for five years (Phase 4 in Fig. 1).

The intensive scaring in Vesterålen is suggested as one of the reasons for an increased use of the staging site in Central Norway, Nord-Trøndelag [31]. In response to this, the Nord-Trøndelag branch of the Farmers’ Union became engaged in the goose issue and increased the pressure on national authorities to work out a solution [37]. In 2004 (Phase 5 in Fig. 1), the Ministry of Agriculture accepted to provide funds for a compensation/subsidy scheme, at first as a pilot project [43]. Sortland was the first municipality where the subsidy was tried out, but the following year, four municipalities in Vesterålen were included; Sortland, Andøy, Hadsel and Øksnes, as well as one municipality further south in Helgeland (Alstadhaug, only barnacle geese at this site). At the same time, four municipalities in Nord-Trøndelag, Steinkjer, Inderøy, Verdal and Levanger, were included. In the preparation for a permanent subsidy scheme, the most difficult issues in the final negotiations between the farmers’ organisations and the ministries of Agriculture and Environment were the source of funding and the question of compensation based on actual damage versus subsidized areas established before goose arrival. The principle of environmental subsidies, entirely funded by the Ministry of Agriculture, was finally accepted by all parties, while the Ministry of Environment would fund necessary research and monitoring. The scheme became a permanent arrangement in 2006. The scheme was based on individual, pre-seasonal contracts where farmers guaranteed that geese were allowed to graze freely on specific fields of cultivated land while receiving a subsidy in return. As a follow-up, a regional management plan for geese in Vesterålen, initiated by the County Governor of Nordland [44], was completed in 2007. The funding for the subsidy program in Norway was 3 million NOKs in 2006 and increased to 3.5 million NOKs in 2007. Practically all farmers who applied and were within a prioritised area received subsidies, although at various rates reflecting the degree of goose densities (assessed by a third party conducting goose counts at a daily basis over the spring staging period). Currently three rates are practiced to accommodate for different goose densities (two rates in 2006–2007).

The number of farms participating in the subsidy scheme in Vesterålen has been relatively stable, with a small increase both in farmers participating and total size of the subsidised areas over the last few years (Table 1). There is a relatively small turnover of individual farmers participating in the scheme, while the most preferred goose sites have consistently been subsidised (unpublished data).

**Table 1:** Number of farms and their sizes in Vesterålen, North Norway.

Year	Number of farms	Total area subsidised area (ha)
2007	71	673.0
2008	76	742.7
2009	77	883.8
2010	76	863.1
2011	82	931.2
2012	72	883.5

The number of farms and the total size of subsidised areas involved in a subsidy scheme for spring staging geese in four municipalities in Vesterålen, North Norway, over the years 2007−2012.

### Trends in Goose Numbers

**Figure 2:**
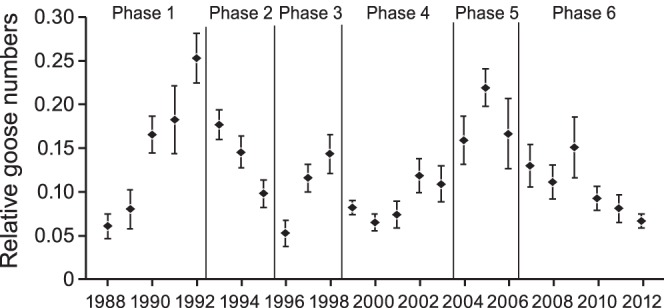
Goose numbers in relative terms based on total population size. The relative number of pink-footed geese *Anser brachyrhynchus* staging in Vesterålen, North Norway, calculated from total population size over the period of 1988–2012. Goose numbers in Vesterålen are yearly averages based on total daily counts within 7–20 May. In 1988−1991 number of count days varied between six and eight days, in 1992–2012 between 11 and 14 days (for all years on average 12.7 days, n = 25 years). Vertical lines are standard errors of the mean.

Over the study period, the pink-footed goose population has increased significantly (Fig. 1, *R^2^* = 0.88, n = 25, *p*<0.0001). The relative numbers in Vesterålen have not changed over the study period (*R^2^* = 0.02, n = 25, *p* = 0.49, Fig. 2), indicating that the local population has increased in correspondence with the total population. The first barnacle geese were registered in 1991 with one pair observed in Sortland municipality on 11 May. In 1994 and 1995, this number had increased to 12 and 28, respectively, and in 1998 barnacle geese were observed in flocks of between 78 and 432 individuals during their staging period. The number of barnacle geese in Vesterålen has increased dramatically thereafter (*R^2^* = 0.84, n = 15, *p*<0.0001) and at present barnacle geese has outnumbered pink-footed geese in this region (Phase 6 in Fig. 1).

### Scaring Activity and Goose Numbers

#### Phase 1; 1988–92, scaring intensity 0

During the first years of the study, Phase 1, there was no scaring by farmers in Vesterålen. There was an abrupt increase in the number of pink-footed geese registered in Vesterålen both in absolute (Fig. 1, *F*_4, 37_ = 9.25, *p* = 0.0001) and relative terms; i.e. number of geese controlled for the total population size (Fig. 1, *F*_4, 37_ = 7.49, *p* = 0.0002).

#### Phase 2; 1993–95, scaring intensity 1

In Phase 2, some farmers started to actively scare geese off their land using different approaches such as tractors, dogs and manual scaring by running, clapping and screaming. Scaring was organised on a neighbourhood basis, especially in Sortland, one of the most affected municipalities in Vesterålen. The response in goose numbers was immediate, with an almost 50% reduction in goosedays from 1993 to 1995 (Fig. 1, *F*_2, 39_ = 4.92, *p* = 0.012). In relative terms, the reduction in goose numbers was also significant (Fig. 1, *F*_2, 39_ = 5.45, *p* = 0.008).

#### Phase 3; 1996–98, scaring intensity 0

Farmers in Vesterålen refrained from scaring in 1996 and the two following years, awaiting implementation of the measures proposed in the Action Plan for Goose Management [41]. The geese responded immediately, with a doubling in goosedays from 1996 to 1998 (Fig. 1, *F*_2, 39_ = 7.53, *p* = 0.002). Also the relative goose numbers increased in this phase (Fig. 1, *F*_2, 39_ = 6.58, *p* = 0.003).

#### Phase 4; 1999–2003, scaring intensity 2

The Action Plan for Goose Management was not immediately implemented, but disputes dragged on between, and within, management agencies, about interpretation of the plan, its formal status, and funding of proposed measures. In 1999, the Sortland branch of the Farmers’ Union decided to resume organised scaring as a reaction to DŃs reluctance to implement compensation to farmers. The scaring activity also increased in other areas in the region (e.g. in the municipality of Andøy), and geese staging in Vesterålen experienced intensive scaring at most sites in this phase. There was a slight increase in goosedays (Fig. 1, *F*_4,65_ = 2.79, *p* = 0.004,), but the relative number of geese using Vesterålen did not increase (Fig. 2, *F*_4, 65_ = 2.18, *p* = 0.08). Barnacle geese established in the region during this phase (Fig. 1), basically utilising the same fields as pink-footed geese for spring staging. Goosedays for barnacle geese increased significantly in this phase, regardless of the intense scaring activity by farmers (Fig. 1, *F*_4, 65_ = 9.69, *p* = 0.0001).

#### Phase 5; 2004–2006, scaring intensity 1

Phase 5 represents the trial period, when subsidies were first introduced in selected areas on a project basis. Scarcity of funds limited the coverage of the program, but there was a substantial reduction of organised scaring in Vesterålen. The pink-footed geese did not, however, respond to this reduction in scaring nor in terms of absolute goosedays (Fig. 1, *F*_2, 37_ = 2.10, *p* = 0.137) nor in terms of relative goose numbers (Fig. 2, *F*_2, 37_ = 1.08, *p* = 0.351), although numbers were higher in 2005 compared to 2004 (Fig. 1, Fig. 2). Barnacle geese, on the other hand, increased significantly over this period of intermediate scaring activity (Fig. 1, *F*_2, 37_ = 22.67, *p* = 0.0001).

#### Phase 6, 2007–2012, scaring intensity 0

While a most of the affected farmers entered the subsidy program in 2006–2007, a small number of farmers have chosen not to apply for subsidies and continue to scare geese off their land. Many farmers have, however, stated that scaring is virtually not necessary as the geese have learned to avoid fields subject to regular scaring (also supported by goose registrations, unpublished data). The geese use the subsidised areas instead, which at present represent the majority of their preferred fields in Vesterålen. The total population size of pink-footed geese increased significantly over this period (Fig. 1) and there were practically no scaring in this six year period. Still, this did not affect the number of pink-footed geese staging in the region as they did not show any increase in neither absolute goosedays (Fig. 1, *F*_6, 75_ = 1.19, *p* = 0.323) nor in relative numbers (Fig. 2, *F*_6, 75_ = 2.02, *p* = 0.074). The amount of barnacle geese on the other hand, increased significantly during this phase (Fig. 1, *F*_6, 75_ = 26.48, *p* = 0.0001).

### The Influence of Scaring and Barnacle Geese on the Relative Number of Pink-footed Geese

**Figure 3:**
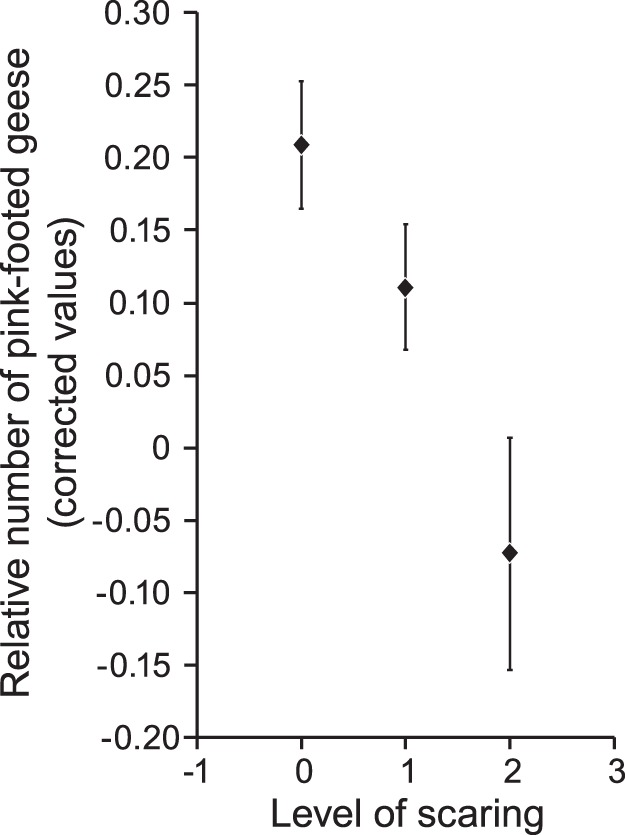
The number of geese and level of scaring. Least Square Means Estimates (see Table 2) for relative numbers of pink-footed geese staging in Vesterålen under different scaring intensities by farmers (0 = no scaring, 1 = moderate scaring, 2 = intensive scaring). Values are corrected for the different phases in the study period (see Fig. 1) and number of barnacle geese staging in the same area. Vertical lines are standard errors.

In addition to the time period (phase), the scaring by farmers and the presence of barnacle geese significantly affected the relative numbers of pink-footed geese (Type 3 Test, Phase: *F* = 5.32, *p* = 0.0001, Scaring activity: *F* = 3.20, *p* = 0.042, Ratio barnacle/pink-footed: *F* = 25.40, *p* = 0.0001). The effect of scaring is shown in Fig. 3, where least square means values illustrate that, regardless of which phase and the numbers of barnacle geese present, intermediate scaring reduced the number of pink-footed geese compared to no scaring, and intensive scaring reduced the number of pink-footed geese even more. In Table 2 the estimates of the different factors are listed. The barnacle goose effects on relative number of pink-footed geese are most pronounced in Phase 6 (lowest estimates).

**Table 2:** The number of geese and estimates of influencing factors.

Effect		Estimate(± stderr)	DF	t-value	P>|t|
Intercept		−0.13 (±0.12)	309	−1.16	0.25
Phase	1	0.02 (±0.02)	309	1.27	0.21
	2	0.09 (±0.08)	309	1.11	0.26
	3	−0.04 (±0.02)	309	−2.43	0.02
	4	0.23 (±0.12)	309	1.99	0.05
	5	0.15 (±0.08)	309	1.81	0.07
	6	0	–	–	–
Scaring	0	0.28 (±0.12)	309	2.42	0.02
	1	0.18 (±0.08)	309	2.23	0.03
	2	0	–	–	–
Ratio barnacle/pink-footed		−0.03 (±0.01)	309	−5.04	<0.0001

Results of a mixed model in which the effect of relative pink-footed goose numbers was estimated in relation to different time periods (Phase 1–6, see Fig. 1), scaring activity by farmers (0 = no scaring, 1 = moderate scaring, 2 = intensive scaring) and barnacle goose numbers relative to pink-footed goose numbers.

## Discussion

The introduction of a subsidy scheme in Vesterålen, North Norway, has reduced the conflicts between geese and agricultural interests to a minimum. At present (2012), very few farmers scare the geese from their properties, and even if there are some scaring activities, the geese have large areas where they can graze undisturbed for the few weeks they stay in the region. The agreement among the main stakeholders is an important success for the scheme, as the immediate justification of the subsidy payment, funded by the agricultural authority, was to resolve the conflict between agriculture and geese. In the Vesterålen case, the process towards an agreement, however, took a considerable amount of time and effort. The main obstacle to reach an agreement at an earlier stage was that the environmental authorities were not willing to fund monetary compensation to farmers. As a compromise, subsidies to farmers were funded by the Ministry of Agriculture, while research and monitoring was funded by the Ministry of Environment. For the affected farmers, it was important to get the recognition that their economic losses were real, the economic burden of hosting geese was skewed towards specific areas and hence unfair, and consequently, a proportional compensation for these losses was necessary. The case has demonstrated the effectiveness of organised scaring, not only to solve immediate problems on individual farms, but also as a means to change the conditions for an entire population of geese and to exert political pressure. Scaring has been part of an organised effort to put pressure on wildlife management policy, and scaring intensity has thus reflected the conflict level during different phases of the process.

Our findings indicate that, with a focus on conflict-solving, it is possible to find a common ground in deliberations between stakeholder groups that enter the process with conflicting values and objectives. Based on the interviews, however, it appears crucial that all parties have confidence in research and monitoring data on the number of geese and the estimated damage to agriculture. The roles of researchers and wildlife managers on national and regional levels are of great importance for the success of the deliberation process. In accordance with the findings by Ormerod and co-workers [45], close and systematic communication and knowledge-transfer between researchers and those who need the knowledge for use, in our case the local agricultural officers at the municipality level, has been a key factor for the positive development of the subsidy scheme in Vesterålen. Furthermore, the coupling of ecological and social science expertises has provided an integrated input in support of alleviation of the conflict, not only quantifying the effects of management actions (or the lack of actions), but also identifying barriers in the decision-making processes [46], [47], [19].

The positive effects of agri-environment schemes, where farmers are financially compensated in order to modify their farming practices in an environmentally advantageous direction, such as accepting geese staging in our study, are often difficult to measure. We assessed the success of the Norwegian subsidy program in North Norway in terms of a) securing acceptable conditions for the geese, b) provisioning of acceptable compensation for damage and c) alleviating the conflict between wildlife and agriculture. The subsidy scheme has demonstrated positive outcome in goose conditions as geese are now able to graze undisturbed in most of their staging areas. The situation of the farmers has also substantially improved, as the subsidies represent an acceptable compensation for economic loss, since most farmers have chosen subsidies instead of a continuation of the scaring activity. The scheme has thus been successful in alleviating the conflict between geese and agriculture, which was extensive in some years in the past. Even if the agricultural community in Vesterålen has been critical to certain aspects of the scheme, the conflict level is currently low, due largely to the active involvement by the farmers’ unions in the process. The fact that the implementation of the subsidy scheme demonstrates a high degree of flexibility and adaptiveness is probably also one of the key factors for success.

Vesterålen is an important spring staging area for the Svalbard population of pink-footed geese. It is their northernmost stopover site before their journey to the breeding grounds in Svalbard. The combination of 24 hours of daylight to feed, early growth of vegetation and behavioural adaptation allowing the geese to utilize the small fields in Vesterålen, provides them with a high energy intake rate [48], [30]. However, in relation to the other sites along the population’s flyway, the area available for geese is limited, meaning that Vesterålen is also a bottleneck for the population [49]. The relatively few farmers involved in the conflict in North Norway will therefore have the potential of influencing the complete flyway and the status of the population due to actions taken at this critical site, e.g. goose scaring [31]. On the other hand, the agricultural activity is important for the region, not only for the farmers involved, but also from a cultural and landscape perspective, hence providing important ecosystem services in the area [50], [51], [52], [53], [54]. In the long-term, sustainability will only be possible with an integration of economic, nature conservation and agricultural landscape interests [55].

The pink-footed geese responded immediately to the scaring activity by farmers. Controlling for both the increase in the population size, the time period (phase) and the presence of barnacle geese showed highly significant differences in numbers among the three categories, i.e., no scaring, intermediate scaring and intensive scaring. Human-induced goose scaring has proved to be successful in influencing the habitat use of wintering geese [56], [18], [57], [10]. Hence, it can be a useful method to reduce the number of geese on prioritised fields. In order to be a successful management tool on a wider scale, however, it is essential to provide alternative feeding areas where the geese can feed undisturbed, which is also essential from a conflict-resolution perspective. The subsidy scheme for spring staging geese in Norway provides such conditions, meaning that the scheme balances population conservation objectives and agricultural interests.

In addition to scaring, the pink-footed geese were significantly influenced by the presence of barnacle geese. Based on winter counts, the total population of barnacle geese has been relatively stable over the last decade [32], [33]. Hence, the considerable increase in Vesterålen must be due to changes in their distribution and not to their population size as such. A gradual northward expansion of spring staging sites has been reported in the early 1990s [34] and it is likely that what we observe today is a continuation of this process. In response to the subsidy scheme, numbers at their most frequently used areas in Helgeland (their traditional spring staging site further south) have also increased [28], [58]. The increase, however, were neither here reflected in their total population size.

The population size of pink-footed geese has increased dramatically over the last decades. On the stopover sites in spring, the trend goes towards an increasing use of Nord-Trøndelag, their staging site in central Norway, and less use of Vesterålen [3, unpublished data, present study]. The presence of barnacle geese in Vesterålen is apparently an important driver of this, causing increased competition for the limited resources (Madsen et al., in prep.). The farmers in the region now experience an increasing number of geese, and, irrespective of the subsidy scheme, they are more concerned about the extent of damage than what goose species is causing it. Barnacle geese tend to be more concentrated close to the coast, and they graze the swards shorter than the pink-footed geese. In consequence, if barnacle geese continue their increase at the expense of pink-footed geese, the pattern of the agricultural damage to farmers is likely to change; with an even more skewed distribution compared to the present situation. A continued monitoring including a close contact between researchers, managers and farmers will be important in order to evaluate how the subsidy scheme needs to be adapted to the dynamic conditions.
